# miRNA-576 Alleviates the Malignant Progression of Atherosclerosis through Downregulating KLF5

**DOI:** 10.1155/2021/5450685

**Published:** 2021-12-08

**Authors:** Jing Wang, Lihui Zhang, Ting Wang, Caige Li, Lijing Jiao, Zhansheng Zhao, Yongjun Li

**Affiliations:** ^1^Department of Endocrinology, The Second Hospital of Hebei Medical University, Shijiazhuang, China; ^2^Department of Cardiology, The Second Hospital of Hebei Medical University, Shijiazhuang, China

## Abstract

**Objective:**

To elucidate the role of microRNA-576 (miRNA-576) in alleviating the deterioration of atherosclerosis (AS) through downregulating krüpple-like factor 5 (KLF5).

**Materials and Methods:**

The AS model in mice was first constructed. Body weight, inflammation degrees, blood lipid, and relative levels of KLF5, miRNA-576, caspase-3, and bcl-2 in AS mice and control mice were compared. Dual-luciferase reporter gene assay was performed to evaluate the binding between miRNA-576 and KLF5. RAW264.7 cells were treated with 200 mg/L ox-LDL for establishing *in vitro* high-fat model. Regulatory effects of miRNA-576/KLF5 on relative levels of *β*-catenin and inflammatory factors in RAW264.7 cells were explored.

**Results:**

Body weight was heavier in AS mice than in controls. Protein levels of KLF5 and caspase-3 were upregulated, while bcl-2 was downregulated in AS mice. In particular, protein level of KLF5 was highly expressed in aortic tissues of AS mice. TC and LDL increased, and HDL decreased in AS mice compared with controls. Inflammatory factor levels were markedly elevated in AS mice. KLF5 was verified to be the target gene binding miRNA-576. Overexpression of miRNA-576 downregulated KLF5, inflammatory factors, and *β*-catenin in ox-LDL-treated RAW264.7 cells. Regulatory effect of miRNA-576 on the release of inflammatory factors in RAW264.7 cells could be partially abolished by KLF5.

**Conclusions:**

miRNA-576 alleviates malignant progression of AS *via* downregulating KLF5.

## 1. Introduction

Atherosclerosis (AS) is one of the most common cardiovascular diseases, posing a serious threat to human health. AS is a chronic progressive disease featured by endothelial damage, lipid deposition, monocyte infiltration, and lipid accumulation and fibrosis in the arteries [1, 2]. AS would lead to other severe cardiovascular lesions [3]. It is important to well elucidate the pathogenesis of AS.

MicroRNAs (miRNAs) are noncoding RNAs with 21-23 nucleotides long, which are extensively distributed in viruses, plants, and metazoans. They participate in posttranscriptional regulation. Abundant evidences have proven the critical role of miRNAs in the progression of AS [4]. They are responsible for regulating the formation and stability of plaques [5–7]. In a recent study, the author uncovered that miR-195-3p alleviates homocysteine-mediated atherosclerosis by targeting IL-31 [8]. Besides, rs41291957 controls miR-143 and miR-145 expression and impacts coronary artery disease risk [9]. It is reported that miRNA-576 is a novel biomarker in renal cell carcinoma [10]. Its specific role in the progression of AS, however, remains unclear.

KLFs (krüpple-like factors) are transcription factors containing zinc finger structure, which are able to mediate DNA transcription. KLF5 is a vital mediator in the occurrence of cardiovascular diseases. It is reported that KLF5 is involved in the development of myocardial hypertrophy and fibrosis, and it could affect the pathological level of myocardial injury through activating the downstream pathways [11, 12]. In vascular smooth muscle cells, miR-9 stimulates cell phenotype changes through targeting KLF5 [13]. In addition, miRNA-152 suppresses malignant progression of AS by downregulating KLF5 [14]. In this paper, we constructed both *in vivo* and *in vitro* AS models, and the regulatory effects of miRNA-576 on the progression of AS were analyzed.

## 2. Materials and Methods

### 2.1. Construction of AS Model in Mice

This study was approved by the Animal Ethics Committee of Hebei Medical University Animal Center. 14 male C57BL/6J ApoE^−/−^ mice (20-25 g) were purchased from Cavens (Changzhou, Jiangsu, China). Mice were habituated in a standard environment with 12 h dark/light cycle, 55-60% humidity, and free access to food and water. They were randomly assigned into two groups, namely, AS group (*n* = 7) and control group (*n* = 7). Mice in the AS group were fed with high-fat diet (HFD) containing 10% custard powder, 10% lard, 1% cholesterol, 78.8% standard diet, and 0.2% sodium taurocholate for 12 consecutive weeks, while those in the control group were fed with standard diet [15].

### 2.2. Cell Culture

RAW264.7 cells were cultured in Roswell Park Memorial Institute 1640 (RPMI 1640) (Gibco, Rockville, MD, USA) containing 10% fetal bovine serum (FBS) (Gibco, Rockville, MD, USA), 100 *μ*g/mL penicillin, and 100 mg/mL streptomycin. Cells were passaged for 4-5 times, and those in good condition were treated with 200 mg/L ox-LDL for 24 h.

### 2.3. Western Blot

Cells were lysed for isolating cellular protein and electrophoresed. Protein samples were loaded on polyvinylidene fluoride (PVDF) membranes (Roche, Basel, Switzerland). Subsequently, nonspecific antigens were blocked in 5% skim milk for 2 hours. Membranes were reacted with primary and secondary antibodies for indicated time. Band exposure and analyses were finally conducted.

### 2.4. Determination of Blood Lipid and Inflammatory Factors

Mice were forbidden to eat overnight before determination. Blood lipid levels and inflammatory factor levels were determined using commercial kits provided by JianKang Technology (Nanjing, China) and Abcam (Cambridge, MA, USA), respectively.

### 2.5. Cell Transfection

RAW264.7 cells in good condition were inoculated in 6-well plates with 2 × 10^5^ cells and transfected using Lipofectamine 2000 (Invitrogen, Carlsbad, CA, USA) at 80% confluence. Fresh medium was replaced at 6-8 h, and medium containing 2 *μ*g/mL puromycin was applied at 48 h. Transfected cells were passaged into a new 6-well plate at 72 h. After cell culture for 1-2 weeks, positive colonies were selected for amplification.

### 2.6. RNA Extraction and Quantitative Real-Time Polymerase Chain Reaction (qRT-PCR)

TRIzol method (Invitrogen, Carlsbad, CA, USA) was applied for isolating cellular RNA. Through reverse transcription of RNA, the extracted complementary deoxyribonucleic acid (cDNA) was used for PCR detection by SYBR Green method. Glyceraldehyde 3-phosphate dehydrogenase (GAPDH) was used as the internal reference. The primer sequences were listed as follows: GAPDH-forward: 5′-CCATGGGGAAGGTGAAGGTC-3′, GAPDH-reverse: 5′-TGATGACCCTTTTGGCTCCC-3′; KLF5-forward (mouse): 5′-CGGCAGTAATGGACACCCTT-3′, KLF5-reverse (mouse): 5′-ATTGTAGCGGCATAGGACGG-3′; U6-forward: 5′-CGCTTCGGCAGCACATATACTAAAATTGGAAC-3′, U6-reverse: 5′-GCTTCACGAATTTGCGTGTCATCCTTGC-3′; and miR-576-forward: 5′-TTGGGTCAAGAGTCAGAAGTTT-3′, miR-576-reverse: 5′-TGGCTTCTACTTGTCCTTTCC-3′.

### 2.7. Dual-Luciferase Reporter Gene Assay

293T cells were inoculated in a 96-well plate with 1.5 × 10^4^ cells. Mutant of plasmids (pGL3-KLF5-MUT and pGL3-KLF5-MUT) was generated by site-directed mutagenesis PCR reaction using platinum pfx DNA polymerase following the product manual. Cells were cotransfected with KLF5 WT/KLF5 MUT and miRNA-576 mimics/negative control, respectively. After transfection for 48 h, cells were lysed for measuring luciferase activity (Promega, Madison, WI, USA).

### 2.8. Statistical Analysis

Statistical Product and Service Solutions (SPSS) 20.0 (IBM, Armonk, NY, USA) was used for all statistical analysis. Data were expressed as mean ± SD (standard deviation). The *t*-test was used for analyzing differences between two groups. One-way ANOVA was enrolled to comprise multigroups. *p* < 0.05 indicated the significant difference.

## 3. Results

### 3.1. KLF5 Was Highly Expressed in AS Mice

**Figure 1:**
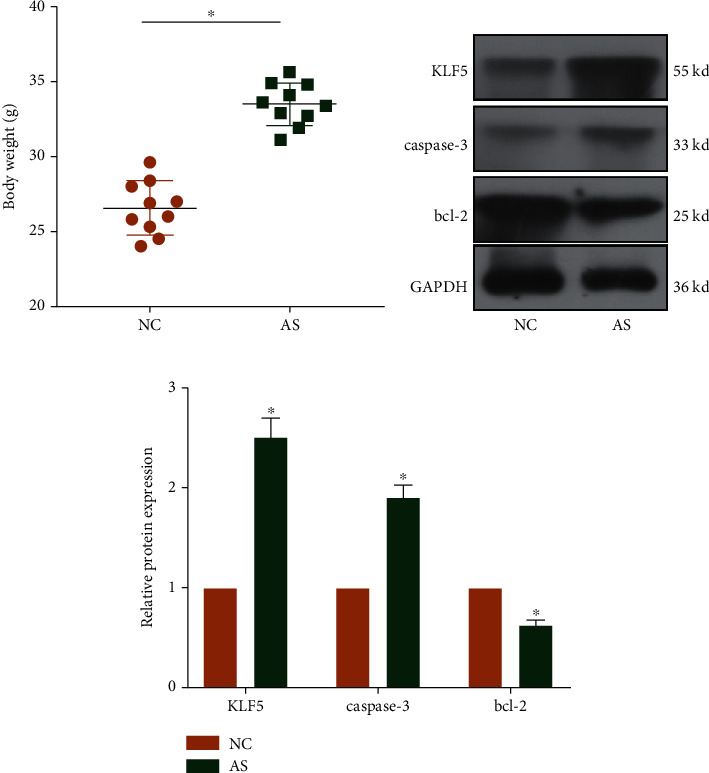
KLF5 was highly expressed in AS mice. (a) Body weight in AS mice and control mice. (b) Protein levels of KLF5, caspase-3, and bcl-2 in AS mice and control mice (c).

Compared with control mice, body weight was heavier in AS mice, suggesting the success construction of *in vivo* AS model (Figure 1(a)). Protein levels of KLF5 and caspase-3 were upregulated, and bcl-2 was downregulated in AS mice (Figure 1(b)).

### 3.2. Relative Levels of Inflammatory Factors and Blood Lipid Increased in AS Mice

**Figure 2:**
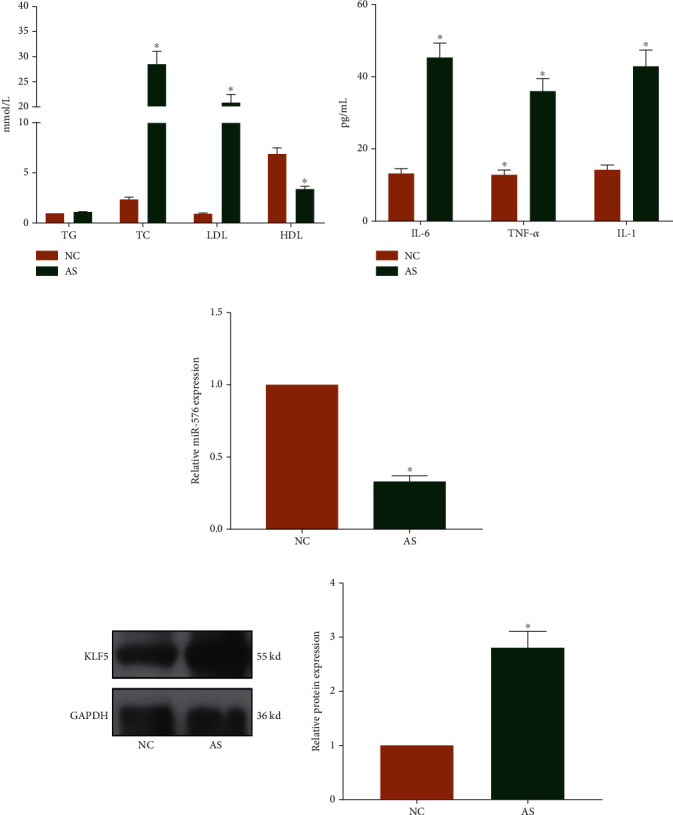
Relative levels of inflammatory factors and blood lipid increased in AS mice. (a) Serum levels of TG, TC, LDL, and HDL in AS mice and control mice. (b) Serum levels of IL-1, IL-6, and TNF-*α* in AS mice and control mice. (c) miRNA-576 level in AS mice and control mice. (d) Protein level of KLF5 in aortic tissues and normal tissues.

Relative levels of TC and LDL increased, and HDL decreased in AS mice, while TG level was similar in AS mice and control mice (Figure 2(a)). Furthermore, serum levels of IL-1, IL-6, and TNF-*α* were elevated in AS mice, verifying the stimulated inflammatory response (Figure 2(b)). Serum level of miRNA-576 was downregulated in AS mice (Figure 2(c)). Western blot analysis uncovered that protein level of KLF5 was markedly upregulated in aortic tissues than that of controls (Figure 2(d)).

### 3.3. KLF5 Was the Target Gene Binding miRNA-576

**Figure 3:**
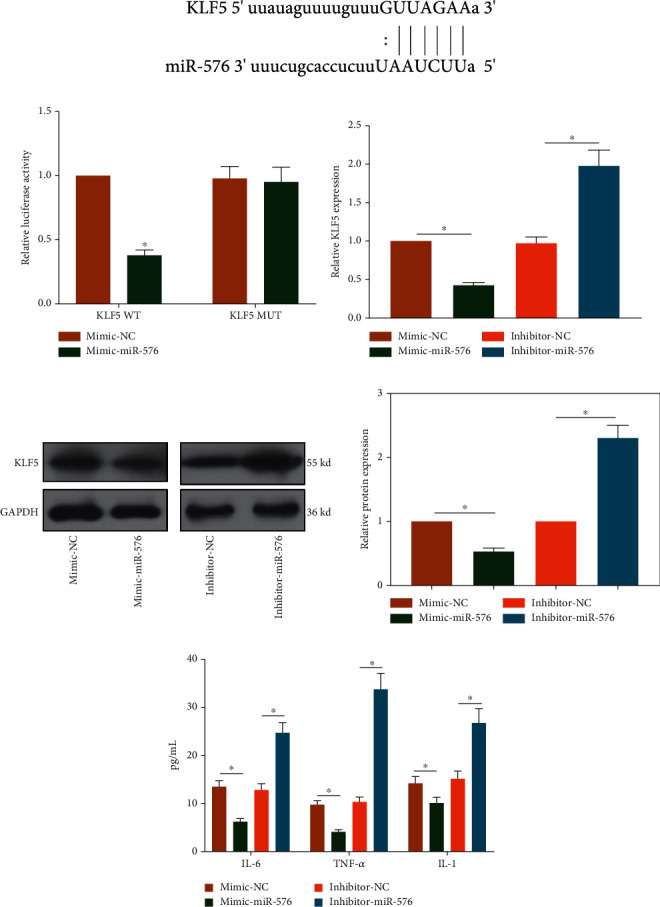
KLF5 was the target gene binding miRNA-576. (a) Binding sequences in 3′UTR of KLF5 and miRNA-576. (b) Luciferase activity in 293T cells cotransfected with KLF5 WT/KLF5 MUT and miRNA-576 mimics/negative control, respectively. RAW264.7 cells were pretreated with 200 mg/L ox-LDL for 24 h. The (c) mRNA and (d) protein levels of KLF5 in RAW264.7 cells transfected with miRNA-576 mimic or inhibitor. (e) Relative levels of IL-1, IL-6, and TNF-*α* in RAW264.7 cells transfected with miRNA-576 mimic or inhibitor.

Through prediction in Starbase3.0, the binding sequences in 3′UTR of miRNA-576 and KLF5 were depicted (Figure 3(a)). Overexpression of miRNA-576 markedly quenched luciferase activity in wild-type KLF5 vector, confirming the binding relationship between miRNA-576 and KLF5 (Figure 3(b)). Subsequently, RAW264.7 cells were pretreated with 200 mg/L ox-LDL for 24 h, and thus, a high-fat microenvironment was constructed. Transfection of miRNA-576 mimic markedly downregulated KLF5, and conversely, transfection of miRNA-576 inhibitor could upregulate KLF5 in ox-LDL-treated RAW264.7 cells (Figures 3(c) and 3(d)). ELISA results demonstrated that overexpression of miRNA-576 markedly downregulated inflammatory factor levels in RAW264.7 cells, and knockdown of miRNA-576 achieved the opposite trends (Figure 3(e)).

### 3.4. *β*-Catenin Was Upregulated in In Vivo and In Vitro AS Models

**Figure 4:**
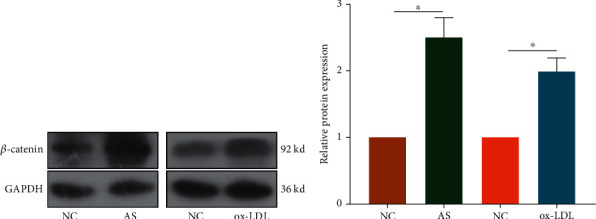
*β*-Catenin was upregulated in *in vivo* and *in vitro* AS models. Protein level of *β*-catenin in AS mice and RAW264.7 cells pretreated with 200 mg/L ox-LDL for 24 h.

Interestingly, the protein level of *β*-catenin was markedly upregulated in AS mice than in controls. In addition, *β*-catenin was identically upregulated in ox-LDL-treated RAW264.7 cells (Figure 4). It is suggested that *β*-catenin may be involved in the pathogenesis of AS.

### 3.5. miRNA-576 Downregulated *β*-Catenin and Suppressed Release of Inflammatory Factors in RAW264.7 Cells through Targeting KLF5

**Figure 5:**
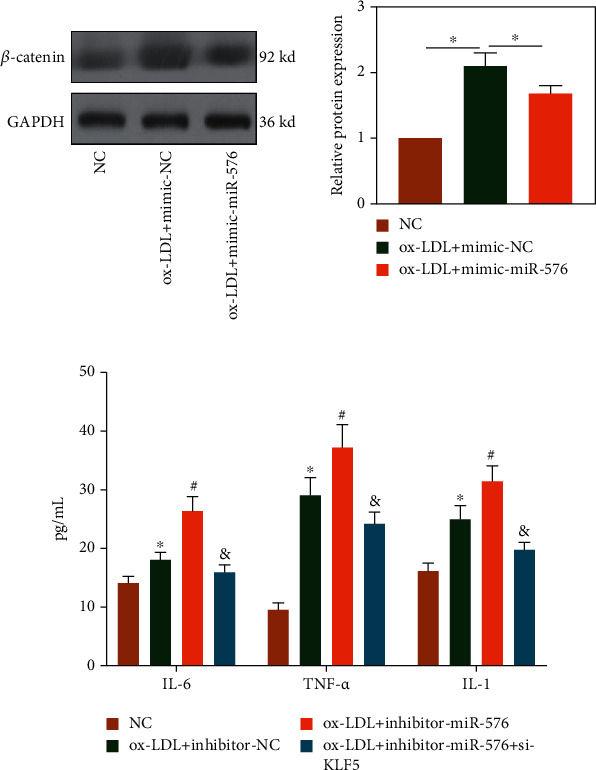
miRNA-576 downregulated *β*-catenin and suppressed release of inflammatory factors in RAW264.7 cells through targeting KLF5. (a) Protein level of *β*-catenin in ox-LDL-treated RAW264.7 cells transfected with miRNA-576 mimics or NC. (b) Relative levels of IL-1, IL-6, and TNF-*α* in ox-LDL-treated RAW264.7 cells transfected with NC, miRNA-576 inhibitor, or miRNA-576 inhibitor+si-KLF5.

The above findings have shown that *β*-catenin was upregulated in ox-LDL-treated RAW264.7 cells. Notably, this upregulated trend was reversed by overexpression of miRNA-576 (Figure 5(a)). Besides, knockdown of miRNA-576 triggered the release of inflammatory factors in RAW264.7 cells, which was abolished by knockdown of KLF5 (Figure 5(b)). Based on our findings, miRNA-576 downregulated *β*-catenin and suppressed release of inflammatory factors in RAW264.7 cells through targeting KLF5, thereafter affecting the progression of AS.

## 4. Discussion

Normal arterial endothelial cells have biological functions of barrier effect, anticoagulation, regulation of vascular tension, and activation of inflammatory mediators [16]. In the process of AS, vascular endothelial cells are impaired by chronic inflammation. Under the coactivation of chemokines and adhesion molecules, inflammatory cells such as monocytes and T cells migrate to the endarterium, where they are differentiated into macrophages. Eventually, monocytes and endothelial cells in the circulation are promoted to migrate and swallow lipids. Thereafter, macrophage foam cells are derived and atheromatous plaques are formed [17]. Multiple miRNAs are involved in chronic inflammatory responses in arterial endothelial cells, thus affecting the development of AS [18].

Wu et al. [19] demonstrated that miR-155 exerts an anti-AS effect on endothelial cells through a negative feedback loop. miR-125a-5p reduces the uptake of oxidized low-density lipoprotein by macrophages through downregulating ORP9. It also inhibits expression levels of inflammatory factors [20–22]. As a DNA-binding transcription factor, KLF5 could bind GT element or CACCC element in the promoter region, thus affecting transcription and expressions of downstream genes. KLF5 is involved in cancer and cardiovascular diseases [23, 24]. In recent years, KLF5 is found to be significant in the progression of AS and cardiac hypertrophy, as well as cell growth of cardiomyocytes and smooth muscle cells [25, 26]. A relevant animal experiment demonstrated that KLF^−/−^ mice present reduced myocardial fibrosis and hypertrophy [27].

In this paper, KLF5 was upregulated and miRNA-576 was downregulated in AS mice. We have verified that KLF5 was the downstream gene binding miRNA-576, and miRNA-576 could negatively regulate KLF5 level. Furthermore, overexpression of miRNA-576 partially reversed ox-LDL-induced upregulation of *β*-catenin in RAW264.7 cells. Knockdown of KLF5 abolished promotive effects of silenced miRNA-576 on the release of inflammatory factors. To sum up, miRNA-576 downregulated *β*-catenin and suppressed release of inflammatory factors in RAW264.7 cells through targeting KLF5, thereafter affecting the progression of AS.

## 5. Conclusions

miRNA-576 alleviates malignant progression of AS *via* downregulating KLF5.

## Data Availability

The datasets used and analyzed during the current study are available from the corresponding author on reasonable request.
